# Addressing contaminants of emerging concern in the United States public water systems

**DOI:** 10.1007/s11356-026-37822-9

**Published:** 2026-05-13

**Authors:** Tasha Siame, Angelique B. Willis, Albert Junior Nyarko, Shno Karimi, Esther Tonade

**Affiliations:** 1 Forestry Department, College of Agriculture and Natural Resources, Michigan State University, East Lansing, MI 48824 USA; 2 Environmental Science and Policy Program, College of Social Science, Michigan State University, East Lansing, MI 48824 USA; 3 Department of Geography, Environment, and Spatial Sciences, College of Social Science, Michigan State University, East Lansing, MI 48824 USA; 4 Department of Community Sustainability, College of Agriculture and Natural Resources, Michigan State University, East Lansing, MI 48824 USA; 5 Department of Communication, College of Communication and Information, University of Kentucky, Lexington, KY 40506 USA

**Keywords:** US drinking water regulation, Policy, Contaminants of emerging concern

## Abstract

Contaminants of emerging concern (CECs) represent a growing yet inadequately governed threat to drinking water safety in the USA. Advances in analytical technologies have detected widespread, low-level contamination from substances like per- and polyfluoroalkyl substances (PFAS), pharmaceuticals, agrochemicals, and personal care products. This raises concerns about the long-term health of humans and ecosystems. Despite growing scientific knowledge, regulatory and policy frameworks have lagged, leading to uneven protections across public water systems. Here, we assess the policy and regulatory infrastructure related to CECs nationwide across all 50 states. Only four states (8%), California, Maine, Wisconsin, and Vermont, had strong, multi-CEC policy infrastructure with enforceable standards, systematic monitoring, and investments in advanced treatment. Many states (36; 72%), distributed across regions, exhibited moderate capacity, typically focusing on PFAS rather than on comprehensive CEC management. In contrast, ten states (20%), mainly in the South and rural West, exhibited poor policy infrastructure with limited regulation, technology, and weak enforcement. These disparities reveal substantial geographic and institutional gaps in drinking water governance. By comparing US and international policies, we propose phased, practical recommendations that combine precautionary regulation, polluter accountability, innovation incentives, and transparency. These steps aim to update drinking water governance to match current chemical challenges, safeguard vulnerable communities, and prepare US water systems for an expanding list of emerging contaminants.

## Introduction

Contaminants of emerging concern (CECs) are pollutants characteristic of the twenty-first century, comprising both natural and synthetic chemicals that have been recently identified in the environment. They can be defined as any physical, biological, or chemical pollutant detected at trace levels in the environment that has potential adverse health or ecological effects, but whose toxicity at low concentrations over prolonged exposure remains poorly understood (Li et al. 2025). CECs remain “emerging” precisely because regulatory standards are only established once harmful effects are confirmed. Despite their potential risks, these substances remain largely unregulated (Hu et al. 2016; Sunderland et al. 2019). Essentially, these include modern-use compounds like industrial “forever chemicals,” including per- and polyfluoroalkyl substances (PFAS), such as perfluorooctanoic acid (PFOA) and perfluorooctanesulfonate (PFOS). They also cover active pharmaceutical ingredients such as antibiotics, hormones, and pain relievers, as well as agrochemicals such as herbicides (e.g., atrazine and glyphosate), microplastics from tire and textile wear, and ingredients from personal care products.

Within this broader category, legacy herbicides like atrazine illustrate how certain regulated contaminants are currently managed under the National Primary Drinking Water Regulations by using running annual averages (USEPA 2006). This approach may not fully capture short-term concentration spikes that exceed health-based thresholds. In contrast, PFAS regulation is increasingly oriented toward maximum contaminant levels to address their persistence and bioaccumulative properties. The USEPA’s recent proposed perchlorate standard (MCLG 20 µg/L) further reflects the continued evolution of regulatory frameworks in response to emerging chemical threats (USEPA 2026c). Regarding personal care products, this may include antimicrobial triclosan in soaps (now removed from antibacterial soaps following FDA action) (FDA 2017), hormone-disrupting preservatives such as parabens, and phthalates used in fragranced products and packaging that may leach into products and water systems. Many of these compounds have been in commercial use for years. However, only recent advances in analytical monitoring have made it clear how extensively they contaminate water and soils (Bexfield et al. 2019; Rahman et al. 2014).

**Fig. 1:**
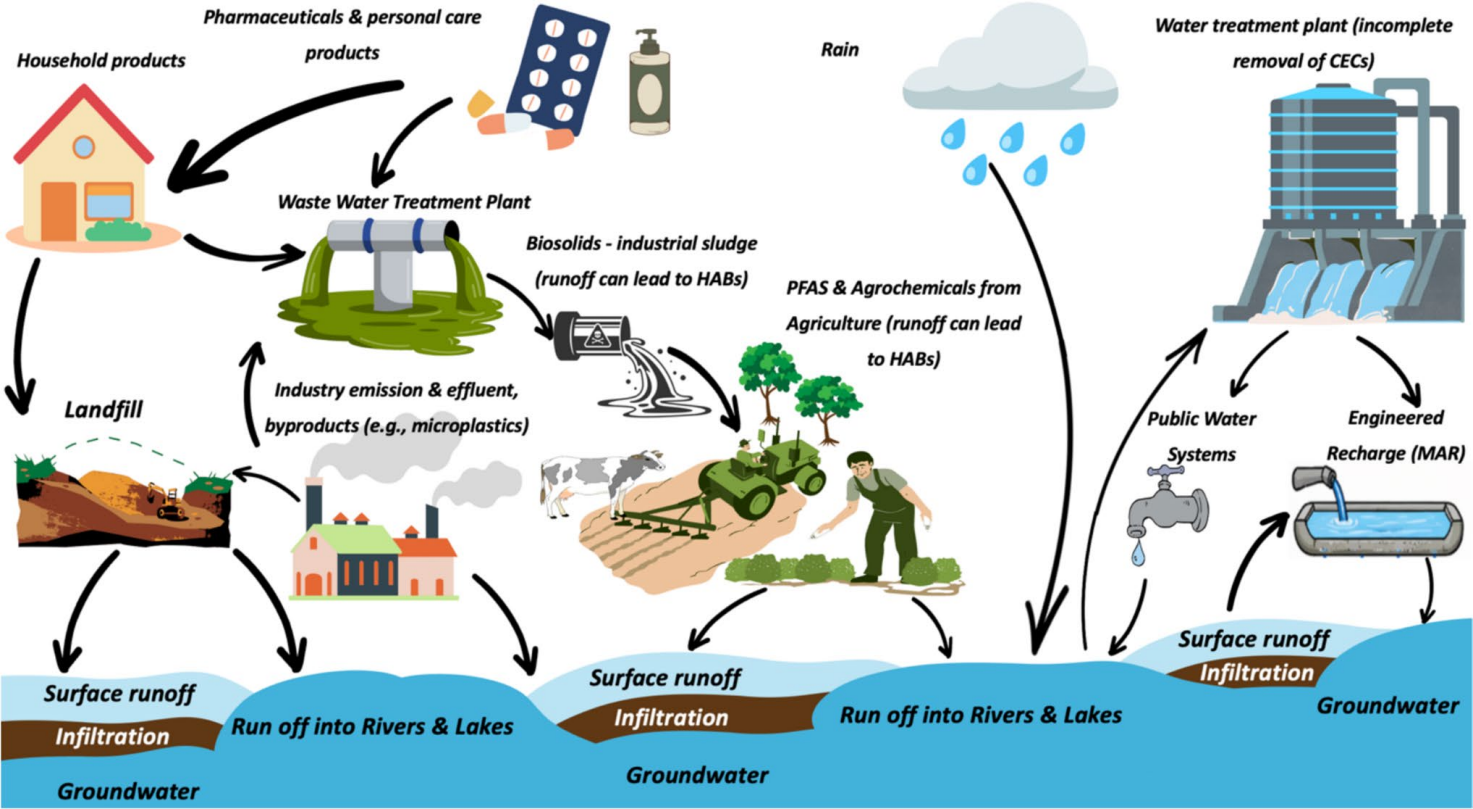
Movement of contaminant of emerging concern (CECs) in the environment to public water systems (graphical presentation drawn using Canva and Microsoft PowerPoint). Note: This figure illustrates the environmental transport pathways of CECs. CECs originate from domestic, industrial, and agricultural sources and enter the water cycle via wastewater discharge, landfill leachate, and agricultural runoff. The runoff from all these sources can lead to harmful algal blooms (HABs). Due to incomplete removal by conventional treatment processes, these compounds can persist in surface water and groundwater, potentially re-entering drinking water systems or recirculating through managed aquifer recharge (MAR). Abbreviations: PFAS, per- and polyfluoroalkyl substances; MAR, managed aquifer recharge; HABs, harmful algal blooms

CECs enter the environment from various sources (refer to Fig. 1), including households through medications, soaps, and cosmetics; agriculture via agrochemicals and manure containing veterinary drugs, and sewage sludge (biosolids) used as nutrient supplements in the USA, where about 53% of wastewater solids are treated and recycled to soils as biosolids fertilizers and soil amendments (NBDP 2018); industry through manufacturing discharges, PFAS, and firefighting foam; as well as stormwater runoff, landfill leachate, and into surface and groundwater (Coderre et al. 2025; NBDP 2018; Siame, et al. 2025a, b). Here, we group examples by chemical class and contamination pathway (e.g., household, agricultural, industrial, wastewater, stormwater (USEPA 2022a). Some substances, such as PFAS or estrogenic compounds, tend to bind to soils and sediments, releasing slowly over time. Drinking-water treatments only partially remove CECs (Xiao et al. 2023). During water treatment, unmonitored transformation products may form, and their risks are poorly assessed. These products may be more persistent, mobile, or toxic than their parent compounds (Rosenblum et al. 2024). The USEPA highlights PFAS, pharmaceuticals, microplastics, and disinfection byproducts as key concerns due to their persistence, toxicity, mobility, and monitoring challenges (USEPA 2022a, 2026b). An analysis of over six million monitoring samples identified inorganic contaminants and disinfection byproducts as the primary health risks, with notable contributions from PFAS and unregulated organics such as 1,4-dioxane and 1,2,3-trichloropropane, emphasizing their regulatory importance (Rosenblum et al. 2024). This risk-based approach builds on national monitoring programs that have elevated PFAS and disinfection byproducts in regulations (Roberson & Eaton 2014). Treatment studies reveal trade-offs, such as activated-carbon removal of PFAS potentially increasing transformation products, which highlights the need to prioritize contaminants across the entire treatment and exposure pathways (Smith et al. 2025). While conventional treatment reduces many contaminants, it is not specifically designed to eliminate trace organic compounds, resulting in treated water that may still contain PFAS, pharmaceuticals, or hormones released into water bodies (Elliott et al. 2025).

New detection technologies, such as high-resolution mass spectrometry (HRMS) and passive samplers, and suspect or non-target screening now reveal trace amounts of CECs in previously overlooked areas, including low concentrations of prescription drugs in rivers and PFAS in rainfall (Pinasseau et al. 2019). These advances improve detection limits and identify transformation products and new contaminants often missed by routine methods. This awareness reveals a policy gap. Although these contaminants are present and enduring, regulations have not kept pace. In the USA, most CECs lack enforceable safety standards, so water utilities are not required to test or limit them (Levin et al. 2024; USEPA 2025a; Xiao et al. 2023). Federal agencies, including the USEPA, Centers for Disease Control and Prevention (CDC), Agency for Toxic Substances and Disease Registry (ATSDR), and United States Geological Survey (USGS) have increasingly recognized the risks posed by CECs through monitoring and assessment. The USEPA advances regulatory oversight through the Unregulated Contaminant Monitoring Rule (UCMR) and the Contaminant Candidate List (CCL) processes; the CDC tracks human exposure via NHANES biomonitoring; ATSDR evaluates public-health risks; and USGS documents environmental occurrence (ATSDR 2021b; CDC 2024; Kingsbury 2022; McMahon et al. 2022; USEPA 2021a).

Under the Safe Drinking Water Act (SDWA), EPA’s (UCMR) and (CCL) processes work together to identify contaminants that might need regulation in the future (USEPA 2021a, 2022a). Large-scale US monitoring revealed that these contaminants persist in drinking-water systems, with detections reported under UCMR 3 and 5 and national USGS surveys finding PFAS, pharmaceuticals, and related compounds in drinking groundwater sources (Kingsbury 2022; McMahon et al. 2022; USEPA 2021a). In response, federal funding through the Bipartisan Infrastructure Law, the Drinking Water State Revolving Fund, and USEPA programs increased support, though costs, proactive policies, and technical barriers persist in many small, tribal, and under-resourced communities (USEPA 2025g, 2025b).

This gap echoes past public health threats such as lead and asbestos, which were regulated only after harm had occurred (Rothschild 2022). Moreover, the Flint water crisis demonstrates how delayed regulatory responses can harm drinking water, erode public trust, and prompt policy changes focused on prevention and accountability (Nelson 2024). Our study addresses this gap by evaluating the effectiveness of current policies, enforcement, and technologies and by proposing policy strategies to reduce CEC levels in public drinking water systems across all 50 US states. In this regard, we focus on unregulated or under-regulated CECs in drinking water and mention legacy contaminants like lead only for context. We further situate our study within the current regulatory context, shaped by the Flint water crisis, growing public concern about drinking water contamination, and ongoing federal or the Office of Management and Budget (OMB) PFAS policy review (ASDWA 2026; Nelson 2024). The main research question we seek to answer is as follows: How can US public water systems address CECs through proactive policy and regulation to ensure safe, equitable, and sustainable access to drinking water?

## Current state of the problem

**Fig. 2:**
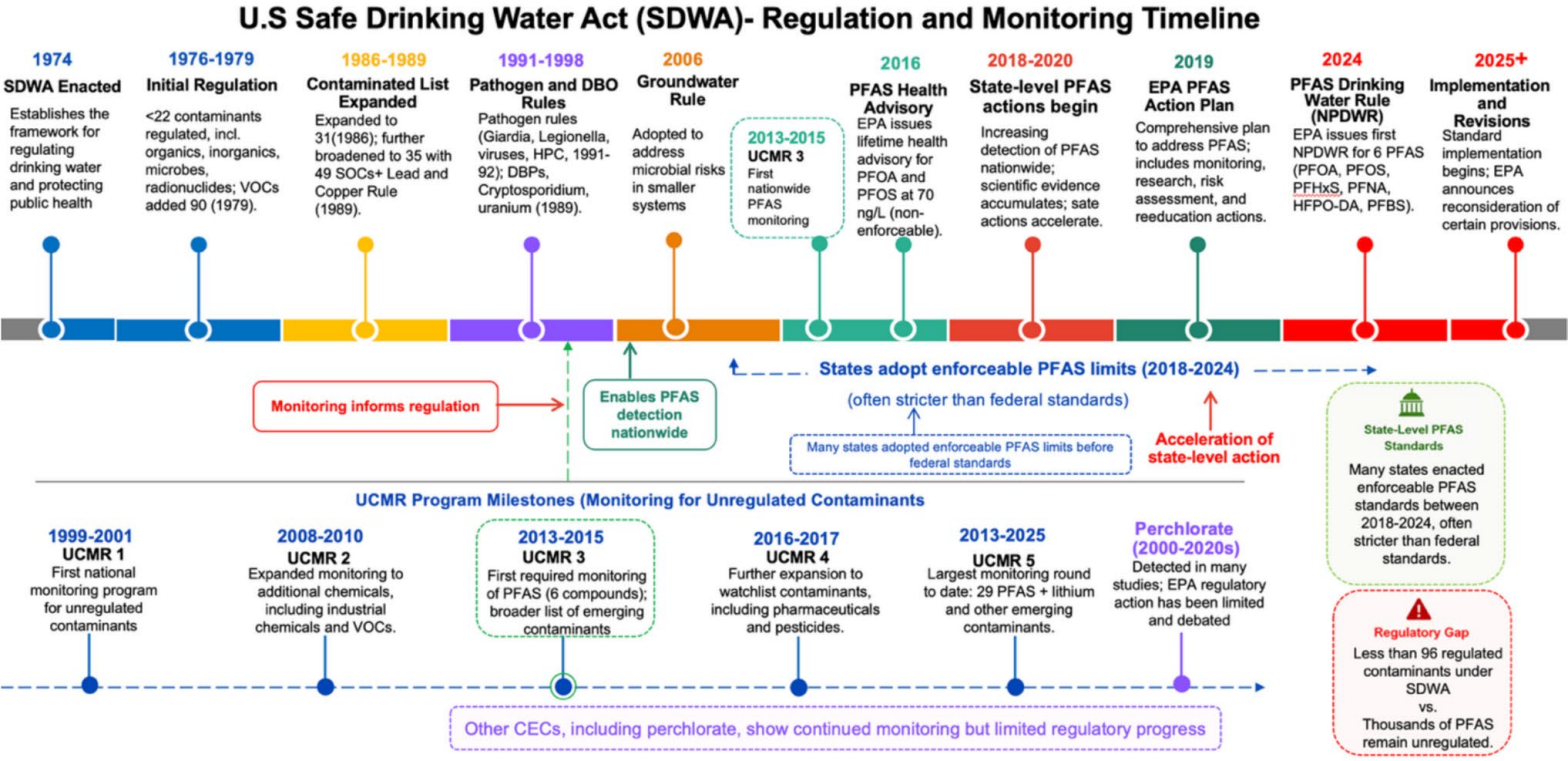
US Safe Drinking Water Act–regulated contaminant of emerging concern (CECs) (timeline drawn using Microsoft PowerPoint). Note: This timeline tracks US drinking water regulation from initial contaminant controls to current attention on PFAS, with policies progressing toward enforceable limits and broader monitoring (USEPA 2025a; Xiao et al. 2023). The abbreviations refer to chemical compounds and water quality terms: PFHxS, perfluorohexane sulfonate; PFBS, perfluorobutane sulfonate; PFNA, perfluorononanoic acid; HFPO-DA, hexafluoropropylene oxide dimer acid, GenX; PFAS, per- and polyfluoroalkyl substances; VOCs, volatile organic compounds; SOCs, synthetic organic compounds; DBPs, disinfection byproducts; HPC, heterotrophic plate count; NPDWR, National Primary Drinking Water Regulation

Currently, the SDWA regulates less than 96 CECs (Refer to Fig. 2). These contaminants are not just hypothetical but are increasingly recognized as real risks in US water systems. They consist of various chemicals that differ in their pathways, persistence, and health impacts, creating a complex risk landscape that traditional water treatment methods and policies are not fully prepared to address. The case of PFAS contamination illustrates this problem. Although six PFAS (PFOA, PFOS, PFHxS, PFNA, HFPO‑DA (GenX), PFBS) are regulated in US drinking water (Evans et al., 2025; USEPA 2025d, 2025e), thousands of other PFAS remain largely unregulated (refer to Fig. 2). Furthermore, a comprehensive nationwide study found PFAS in 194 water sources across 33 states, with 66 systems exceeding the EPA’s 70 ng/L advisory level, first issued on January 8, 2016 (Hu et al. 2016). These figures likely underestimate the true extent, as many smaller and tribal systems were not sampled. Due to their persistence, these chemicals accumulate in the environment and human bodies, where they are associated with immune suppression, cancers, thyroid issues, and decreased vaccine effectiveness in children (Grandjean et al. 2012; Levin et al. 2024; Sunderland et al. 2019). Additionally, the EPA’s recent proposal to set enforceable limits at 4 ng/L for PFOA and PFOS, issued on April 10, 2024, 8 years after the January 8, 2016, advisory, reflects how emerging science has surpassed previous guidelines (USEPA 2024b).

Additionally, pharmaceuticals are increasingly detected in water sources, raising important environmental and public health concerns. Studies have identified medications such as carbamazepine, diclofenac, and various antibiotics downstream of wastewater treatment facilities, often surviving conventional treatment processes (Aus der Beek et al. 2015; Coderre et al. 2025; Schaider et al. 2014). The primary concern is not acute toxicity, but rather chronic, low-level exposure and its broader ecological implications. For instance, residues of synthetic estrogens have been associated with endocrine disruption in aquatic organisms, with feminization effects observed in fish populations downstream of treatment plants (Kidd et al. 2007; USEPA 2025a). The ecological disturbances caused by trace chemical presence may lead to adverse human health outcomes, posing risks to human health (Vandenberg et al. 2012).

In agriculture, chemical contaminants such as agrochemicals introduce additional complexities. Neonicotinoids, including imidacloprid and clothianidin, are widely utilized in US agriculture and have been detected in drinking water at trace concentrations ranging from 0.24 to 57.3 ng/L (Klarich et al. 2017). Furthermore, atrazine, a prevalent legacy herbicide, remains detectable in groundwater and surface water, with documented associations to developmental toxicity and endocrine disruption (Hayes et al. 2011). These biologically active agrochemicals pose risks to neurological and reproductive health, and their high mobility in soils, alongside their persistence in aquatic systems, renders rural populations reliant on shallow aquifers particularly vulnerable (Uychutin et al. 2024).

Moving forward, chemicals like ticlosan and bisphenol A in personal care products contaminate groundwater and surface water, acting as endocrine disruptors and forming disinfection byproducts during water treatment (Bexfield et al. 2019; Halden 2014; Richardson & Ternes 2018). Long-term low-dose exposure, especially in mixtures, remains poorly understood (Vandenberg et al. 2012). Beyond the science, exposure disparities reveal vulnerabilities in the US drinking water system. Small rural utilities lack resources for advanced treatment, leaving communities at risk (USEPA 2025a; Xiao et al. 2023). Private wells, which serve 15% of Americans, are unregulated and mainly affect low-income and rural households (ATSDR 2021a; Balazs & Ray 2014). Additionally, racial and socioeconomic disparities persist, as seen in Flint, Michigan, where residents faced regulatory failures (Hanna-Attisha et al. 2016).

It is essential to recognize that the consequences of inaction are significant. As highlighted by Trasande et al. (2024), failing to address exposure to CECs could incur costs totaling billions of dollars, equivalent to more than 1% of gross domestic product (GDP). Exposure to PFAS alone, for example, can lead to health issues such as increased risks of kidney and testicular cancers, immune system problems, and metabolic disorders (Grandjean et al. 2012; Steenland et al. 2013). Additionally, pharmaceuticals and agrochemicals cause ecological harm, from endocrine disruption in fish to antibiotic resistance in microbes (Gkotsis et al. 2022; Levin et al. 2024). Microplastics and other CECs may also biomagnify across food webs, resulting in higher concentrations in predators and potentially greater human exposure through feed-to-food pathways (Gkotsis et al. 2022; Kidd et al. 2007; X. Li et al. 2023). Equally important, public trust in water systems declines when unregulated contaminants are detected, thereby reducing confidence in utilities and regulators (Rahman et al. 2014). Globally, the US lags behind Europe, where the EU maintains a “watch list” of emerging pollutants and actively sets limits (Xiao et al. 2023). Without intentional and continuous reform, US systems may continue to lag in delivering safe and equitable water services.

Overall, scientific evidence indicates that CECs are present and pose risks to health and trust (Grandjean et al. 2012; Levin et al. 2024; Xiao et al. 2023). Addressing this issue requires proactive regulation, investment in treatment technologies, and specific protection of vulnerable communities. It also requires early warning systems that utilize high-resolution mass spectrometry-based non-target screening to identify previously unrecognized contaminants, including “unknown unknown” such as newly synthesized or non-registered chemicals already present in the environment (Alygizakis et al. 2018; Paszkiewicz et al. 2022). Thus, understanding the problem’s scope is only the first step; the more urgent task now is to translate scientific findings into policies that ensure safe drinking water for everyone.

## Methods

Our study conducted a comparative policy assessment to measure progress in managing CECs across all 50 US states. We systematically reviewed state policies, regulatory programs, and enforcement mechanisms related to drinking water and source water protection, with a focus on CECs, including PFAS, pharmaceuticals, agrochemicals, and personal care products (refer to Supplementary Table 1). Data were collected from federal and state regulatory databases, including EPA documentation, state environmental agency reports, and publicly accessible policy records, following methods used in previous cross-country and cross-state environmental policy evaluations that combine multiple sources (Chidiac et al. 2023; De Castro-Pardo et al. 2023; Dulio et al. 2018).

States were assessed using a three-criterion framework that captures regulatory intent and capacity for implementation: (1) the existence and enforcement of policies and programs related to CECs (standards, monitoring mandates, compliance mechanisms); (2) the adoption of advanced treatment or monitoring technologies, including state-supported innovation efforts; and (3) an overall composite score reflecting policy, enforcement, and technological readiness, aligned with best practices for environmental governance indices (Chidiac et al. 2023; De Castro-Pardo et al. 2023). Each state was categorized on a 3-point scale: poor (little or no formal CEC policy infrastructure), moderate (focused actions targeting specific CEC classes or pilot projects), and strong (comprehensive, multi-CEC regulatory and technological frameworks) (refer to Supplementary Table 1).

## Results and discussion

### Policy options

**Fig. 3:**
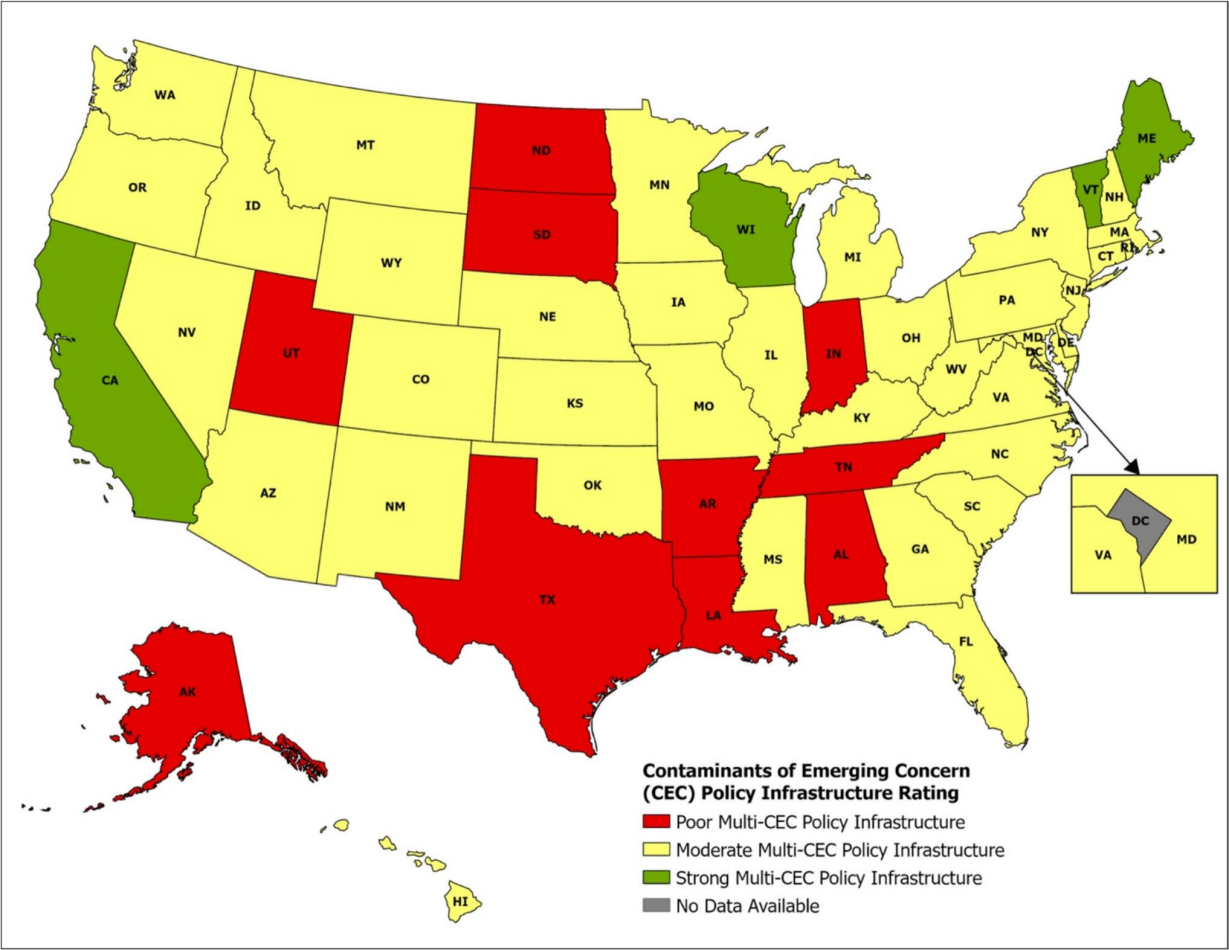
US state-level multi-contaminant of emerging concern (CEC) policy infrastructure ratings (map created using ArcGIS Pro). Note: States were classified using a three-criterion assessment of regulatory frameworks, enforcement capacity, and technological readiness for managing CECs. The resulting categories, poor, moderate, and strong, reflect relative differences in policy comprehensiveness and implementation among states (refer to “Methods” section and Supplementary Table 1 for details). The District of Columbia is marked as “No Data Available” not because of a lack of drinking water oversight, but because USEPA Region 3 administers oversight rather than a state-run primacy program (refer to “Methods” section). As a result, DC does not align with the state-based policy-scoring framework used in this analysis

Our assessment of the national policy landscape revealed substantial differences in how states are equipped to handle CECs. Out of 50 US states evaluated, only four (8%), California, Wisconsin, Maine, and Vermont, were identified as having a strong multi-CEC policy infrastructure, featuring enforceable standards, ongoing monitoring, and investments in advanced treatment or innovation (refer to Fig. 3 and Supplementary Table 1). The majority, 36 states (72%), were categorized as moderate, indicating partial or contaminant-specific measures, primarily targeting PFAS, with less emphasis on pharmaceuticals, agrochemicals, or personal care products. In contrast, 10 states (20%) were deemed poor, showing limited regulatory coverage, weak enforcement, or dependence on voluntary or pilot programs (Fig. 3 and Supplementary Table 1). Across the four states classified as strong, the commonality was not a single identical law, but a layered policy structure that combined enforceable contaminant standards or action thresholds, recurring monitoring, and state-supported treatment or innovation capacity (Fig. 3; Supplementary Table 1).

In this sense, California, Wisconsin, Maine, and Vermont stood out because they moved beyond narrow, single-contaminant responses and demonstrated a broader capacity to link regulation, surveillance, and implementation. These approaches could, in principle, be adapted by other states or scaled to the federal level through minimum national monitoring requirements, provisional class-based benchmarks, and dedicated support for treatment upgrades. However, broader adoption would still depend on uneven state capacity, laboratory access, and sustained financing (Balazs & Ray 2014; Levin et al. 2024; Xiao et al. 2023). As shown in Fig. 3, the District of Columbia is classified as “No Data Available” not because drinking water oversight is absent, but because oversight is administered by EPA Region 3 rather than through a state-run primacy program. As a result, DC did not fit the state-based policy-scoring framework applied in this analysis.

These findings support earlier policy assessments indicating that US chemical governance remains largely reactive, fragmented, and varies across jurisdictions (Levin et al. 2024; Nwokediegwu et al. 2024; Xiao et al. 2023). The DC was excluded due to its unique regulatory status, which lacks the independent state-level policy documentation and reporting needed for this comparison, an issue also noted in previous governance comparison studies (Dulio et al. 2018; Puri et al. 2023). The geographic spread of policy capacity further implies that states with fewer institutional resources are less likely to adopt comprehensive CEC strategies, reinforcing patterns of regulatory inequality seen in environmental health research (Chidiac et al. 2023; Levin et al. 2024; Nwokediegwu et al. 2024). Overall, these findings indicate that relying solely on state-led initiatives has led to an inconsistent national approach, leaving many populations unprotected from emerging chemical threats. This pattern provides a useful basis for considering policy options that can improve regulatory consistency, reduce disparities, and help US water governance become more precautionary and proactive.

### Precautionary regulation

A precautionary regulatory framework is one of the most widely advocated approaches for addressing CECs. Rooted in the principle of acting in the face of scientific uncertainty, precautionary regulation acknowledges that delaying interventions until complete causal certainty is established can result in irreversible harm (Nakayama et al. 2019; Panieri et al. 2022; Tickner et al. 2003). This approach has been adopted in the European Union (EU) through watch-list and risk-based mechanisms embedded in recent water policy, including Union-wide monitoring lists under the Water Framework Directive and a drinking-water watch list under Directive (EU) 2020/2184 (Dettori et al. 2022; Schreiber et al. 2024). By contrast, the USA has often advanced through contaminant-specific rulemaking under the SDWA. That distinction should not be overstated. Legacy contaminants such as atrazine are already regulated under the National Primary Drinking Water Regulations, and USEPA has now established legally enforceable drinking water standards for six PFAS. Even so, the US framework remains more contaminant-by-contaminant, whereas the EU approach relies more explicitly on periodic watchlist updates and risk-based monitoring to identify substances for future action. Recent US support for compliance has also expanded through the Infrastructure Investment and Jobs Act and the Emerging Contaminants in Small or Disadvantaged Communities grant program (USEPA 2025a). However, a direct comparison with Europe is less straightforward because implementation support under Directive (EU) 2020/2184 is largely carried out through member states and can be adapted to national and geographic circumstances, rather than through a single EU-wide funding pathway for small utilities (Schreiber et al. 2024; USEPA 2025a).

Thus, a practical way to embed precaution into the SDWA framework would be to authorize provisional, class-based health benchmarks or trigger values for priority CEC groups, coupled with targeted monitoring, public notification, and scheduled reevaluation as toxicological evidence evolves. States already provide useful analogs for this kind of approach. Under the SDWA, states may set and enforce drinking water standards so long as they are at least as stringent as EPA’s national standards. California, for example, uses notification and response levels for chemicals that do not yet have MCLs, including PFAS and cyanotoxins. Maine implemented an interim PFAS drinking water standard that required sampling, customer notification, and remedies when exceeded, and Massachusetts adopted a state PFAS MCL before the recent federal PFAS rule. Taken together, these examples suggest that precaution need not wait for a fully matured federal rulemaking cycle. It can be operationalized through interim thresholds, required monitoring, communication obligations, and periodic updating as evidence strengthens (CSWRCB 2025; MCDC 2025; MDEP 2020; USEPA 2026e).

For PFAS specifically, the challenge is not that they are wholly unregulated, but that thousands of PFAS exist and regulating them one by one is impractical, even as EPA has already moved from advisory levels to legally enforceable drinking water standards for six PFAS (USEPA 2025e, 2025d). These provisional standards could be revisited on a scheduled cycle (e.g., every 5 years), ensuring that regulation keeps pace with toxicological advances. Such a tiered system would provide legal certainty for utilities, predictable protection for communities, and regulatory flexibility for emerging contaminants where evidence is still evolving. Scholars argue that precautionary standards are particularly important in contexts of environmental justice, where communities historically subjected to disinvestment and regulatory neglect cannot afford further delays in protection (Hanna-Attisha et al. 2016; Siame et al. 2025a, b). In this sense, precaution is a policy imperative to avoid replicating past failures in which regulatory inaction led to irreversible harm, as with lead and asbestos (Rothschild 2022).

### Polluter pays principle

Operationalizing the polluter-pays principle for CECs presents another policy avenue, representing a significant shift in how US drinking water contamination is managed. Under the current system, the financial burden of testing and treatment overwhelmingly falls on utilities and, by extension, ratepayers (USEPA 2025h). This model disproportionately harms small and rural systems, which make up the majority of US public water suppliers and often lack the fiscal capacity to finance advanced treatment technologies (Balazs & Ray 2014; USEPA 2025a). By contrast, the polluter pays principle would assign liability to the industries that profit from chemical production, thereby addressing both environmental risk and distributive justice.

There are several pathways for integrating this principle into CEC governance. One is strict liability frameworks modeled after the Comprehensive Environmental Response, Compensation, and Liability Act (CERCLA), which already holds polluters accountable for hazardous waste sites (USEPA 2025j). While CERCLA has historically targeted localized contamination hotspots, expanding its scope to include diffuse waterborne CECs could compel chemical manufacturers and pharmaceutical companies to finance monitoring, remediation, and long-term health surveillance. CERCLA’s clearest strengths lie in site-specific contamination cases involving identifiable responsible parties, where the statute supports cleanup authority, cost recovery, and, when needed, alternative water supplies for affected households. Legacy groundwater contamination by chlorinated solvents such as trichloroethylene (TCE) and perchloroethylene (PCE) illustrates this point, as Superfund remedies have long included treatment and alternate water supply responses for contaminated drinking water sources (USEPA 2025g). For private well owners in particular, this is an important feature, because CERCLA response actions may include replacement drinking water or household water supplies where contamination can be tied to a specific site or release.

For example, several states, including Minnesota, Michigan, and New Jersey, have pursued litigation against PFAS manufacturers, resulting in billions of dollars in settlements earmarked for cleanup and treatment (Safer States 2023). A particularly instructive example is the Chemours consent order in North Carolina, which stands out because it tied liability to a dominant, identifiable facility and required not only investigation and reduction of PFAS releases, but also private well sampling, replacement drinking water supplies for affected residents, ongoing disclosure of previously undisclosed PFAS, and characterization of PFAS mass loading to downstream water supplies. This type of precedent may be more transferable to other CECs where a major source can be clearly identified and linked to drinking water contamination, but it is less easily extended to diffuse pollutants or cases in which liability is spread across multiple compounds, actors, or pathways (NCDEQ 2019). Translating such litigation-driven outcomes into a federal cost-recovery program would ensure uniformity and reduce reliance on states’ uneven capacity to pursue industry accountability.

At the same time, CERCLA also provides an important cautionary lesson. After the Superfund tax authority expired at the end of 1995, the program lost its dedicated industry-based funding stream until taxes were reinstated in 2021, with the reinstated taxes taking effect in 2022 (GAO 2025). In addition, EPA recovered $155 million from potentially responsible parties in FY2021 and $97 million in FY2022. These recoveries also depended on substantial federal enforcement capacity, including $17.5 million and $17.2 million in budgeted Superfund reimbursements to the Department of Justice’s Environment and Natural Resources Division in those same fiscal years (USDJ 2024). Earlier GAO evidence further showed that when responsible parties refused to settle, litigation over access and information could delay cleanup and the identification of other responsible parties for nearly five years (GAO 2009). For that reason, any CEC framework modeled in part on CERCLA should be designed to avoid these weaknesses by pairing polluter liability with uninterrupted industry financing, streamlined administrative settlement pathways, and dedicated enforcement resources so that monitoring and treatment do not depend primarily on prolonged case-by-case litigation (GAO 2009, 2025; USDJ 2024).

Another option involves establishing producer responsibility fees levied on the sale of specific chemical classes (e.g., PFAS, neonicotinoids, or synthetic pharmaceuticals) and directing these revenues into a national CEC remediation and innovation fund. Similar models exist in other policy domains. Extended producer responsibility (EPR) laws have been applied to electronics, packaging waste, and pharmaceuticals in Europe and Canada, requiring manufacturers to finance the end-of-life management of their products (Tumu et al. 2023). Applying EPR to water pollutants would create an economic disincentive for reliance on persistent and bioaccumulative compounds while generating resources for utilities to invest in advanced treatment technologies. Importantly, fee structures could be designed to scale with production volumes, toxicity, or persistence, thereby incentivizing companies to transition toward safer alternatives (Sharma et al. 2025). The benefits of this approach are that it can shift part of the financial burden away from utilities and ratepayers, generate dedicated producer funding, and encourage upstream product redesign. However, EPR systems also have limitations, including administrative complexity, uneven incentives in collective schemes, and the risk that poorly designed fee structures will raise revenue without meaningfully changing producer behavior. One practical option would be to collect these fees into a dedicated national CEC remediation fund rather than a general fund. The distribution would be administered through states, tribes, or territories using a priority-based framework analogous to the Drinking Water State Revolving Fund, which already channels federal support through state programs that rank projects based on health risk, compliance needs, and system affordability (OECD 2016). Under such a model, revenues could be earmarked specifically for eligible public water systems facing documented CEC burdens, treatment needs, or source-water contamination, thereby helping ensure that funds are directed to impacted systems rather than absorbed into broader budgetary uses.

### Innovation incentives

Innovation incentives offer another forward-looking policy pathway that addresses both the prevention of new contaminants and the remediation of existing ones. Traditional “end-of-pipe” technologies, designed to strip contaminants out of water after contamination has already occurred, remain critical but are inherently reactive and cannot keep pace with the chemical diversity of modern society (Rahman et al. 2014). Research on advanced adsorbents, nanofiltration, ion exchange resins, and biochar-based materials has shown promising results for the removal of PFAS, pharmaceuticals, and agrochemicals, often achieving efficiencies that exceed those of conventional activated carbon (Ross et al. 2018; J. Wang & Wang 2016). Additionally, the literature makes clear that technology choice should be tailored to contaminant properties rather than treated as universally interchangeable. For example, PFAS-specific anion exchange resins can be especially effective for many negatively charged PFAS, whereas activated carbon often provides broader removal of diverse organic micropollutants. Thus, the most appropriate treatment train depends on whether utilities are targeting a narrow class of contaminants or a wider mixture of compounds with different charge, polarity, and mobility characteristics (Aumeier et al. 2023; Rückbeil et al. 2025).

However, barriers to widespread adoption include high operational costs, energy demands, and scalability challenges (Rahman et al. 2014). Moreover, non-destructive technologies such as adsorption, ion exchange, and membrane separation do not eliminate contaminants. Instead, they concentrate them into spent media, brines, or residuals that can become secondary environmental sources if they are not properly regenerated, destroyed, or disposed of (Blotevogel et al. 2023; USEPA 2024a). In contrast, destructive technologies are attractive because they seek complete or near-complete mineralization of organic contaminants rather than simple phase transfer, but many remain energy-intensive, matrix-sensitive, or insufficiently mature for routine full-scale use, especially outside source-specific PFAS applications (Blotevogel et al. 2023; USEPA 2024a). Federal and state governments could accelerate deployment through targeted grant programs, tax credits, and public-private partnerships, paralleling policy tools that stimulated renewable energy technologies and electric vehicles (Sovacool 2009). Current EPA efforts already move in this direction through the Emerging Contaminants in Small or Disadvantaged Communities grant program and the Tackling Emerging Contaminants initiative, which support treatment, testing, and technical assistance for small or disadvantaged systems. However, although these programs allow projects addressing contaminants on USEPA’s CCL, current implementation and guidance remain strongly centered on PFAS, and USEPA’s Office of Inspector General has noted that the agency has provided less clarity on how non-PFAS emerging contaminant projects should be ranked and funded. This suggests that future program design should more explicitly consider whether additional CEC groups, beyond PFAS, warrant stronger prioritization where states document meaningful public health concerns (USEPA 2025c, 2026a, 2026d).

In this context, an equity lens is essential in designing these incentives. Without targeted funding structures, innovation in treatment technologies risks being concentrated in large, urban utilities, while small rural systems, already facing the highest exposure burdens, remain excluded from pilot testing and infrastructure modernization (Rahman et al. 2014). Mechanisms such as set-aside funds for disadvantaged systems, federally managed demonstration projects, and technical assistance programs could help ensure that the benefits of innovation are distributed more equitably. In this way, innovation incentives would aim to structure innovation systems to address both efficiency and justice in drinking water governance.

### Community right-to-know and transparency

Currently, utilities are required to produce annual Consumer Confidence Reports (CCRs), but these reports cover only regulated contaminants and are often written in technical language that is inaccessible to most residents (Johnson 2000). Recent revisions to the CCR framework and existing public notification requirements partially address these concerns, but they do not fully resolve the broader issue that information on unregulated contaminants may still be difficult for communities to access, interpret, and use. As a result, even when water quality information is available, its format and scope limit its utility for affected communities. This opacity has been repeatedly cited as a driver of public distrust, particularly in communities of color and economically disadvantaged areas that are disproportionately affected by regulatory failures, such as the Flint water crisis (Hanna-Attisha et al. 2016; Pulido 2016; Ranganathan 2016).

A noteworthy example of the power of transparency is the US Toxics Release Inventory (TRI), which demonstrates how public disclosure of industrial emissions can increase community access to information and incentivize corporate reductions in toxic releases (Chartres et al. 2022). Translating this model to drinking water governance would mean mandatory disclosure of CEC monitoring results in publicly accessible formats, such as geospatial databases, interactive dashboards, and mobile applications. Nonetheless, such transparency mechanisms cannot assume that all utilities have the same capacity to monitor, manage, and publish data. Smaller rural and tribal systems often face substantial technical, managerial, financial, staffing, and equipment constraints. This limits contaminant testing, data entry, and long-term digital maintenance, making stand-alone dashboard or app-based disclosure requirements potentially cost-prohibitive and inequitable (Balazs & Ray 2014; USEPA 2021b, 2025f). Mandatory disclosure also needs careful design to avoid discouraging voluntary monitoring or exploratory research on unregulated CECs. This is especially important when results come from newer or non-standard analytical approaches, because such findings may require additional qualification, validation, and plain-language explanation before they can be responsibly communicated to the public. In some cases, federal records may also be requested through the Freedom of Information Act.

A more feasible approach would be to place the primary responsibility for digital infrastructure at the state or federal level through centralized reporting portals, standardized templates, shared laboratory support, and dedicated technical assistance so that transparency does not become an unfunded mandate for under-resourced systems (USEPA 2021b, 2025f). Such tools could allow residents to track exposures by zip code or service area, thereby democratizing access to information that utilities and regulators have historically controlled. Current federal monitoring frameworks, such as the UCMR, illustrate both the potential and the limitations of existing approaches. While UCMR has generated valuable national data on select CECs, participation is structured by utility size rather than a single cutoff. All large community and non-transient non-community systems serving more than 10,000 people are required to monitor, while systems serving 3300 to 10,000 people are identified separately because they represent a smaller-system category newly brought into full UCMR 5 monitoring under the America’s Water Infrastructure Act, subject to available appropriations and sufficient laboratory capacity. Only a statistically representative sample of systems serving fewer than 3300 people is included (USEPA 2025i).

Importantly, UCMR is intended to generate occurrence data on unregulated contaminants to inform future regulatory decisions, rather than to function as universal surveillance for every utility and every emerging analytical method. In that sense, the current model largely fulfills its statutory role. However, if the goal is broader public health protection, further adjustments may still be warranted. Expanded monitoring can be burdensome for utilities, especially small, rural, tribal, and remote systems, because sampling schedules, transport timelines, hold times, laboratory coordination, and data reporting requirements can be difficult to manage in under-resourced settings. For systems with low expected vulnerability, an opt-in or more targeted supplemental monitoring approach may be more practical than universal expansion, particularly for newer analytical approaches that are not yet standardized for routine compliance use.

As a result, many rural and tribal communities are excluded from monitoring altogether, leaving significant geographic gaps in exposure information. These omissions highlight the need for universal, publicly accessible disclosure requirements that extend beyond the scope of UCMR to cover all utilities, regardless of size, thereby ensuring that rural and tribal communities are not left invisible in national monitoring efforts.

### US and global policy landscape comparisons

Global governance of water quality and chemical pollution has evolved substantially in response to growing concerns about CECs (refer to Table 1). International organizations, regional blocs, and national governments now implement a diverse array of regulatory and policy frameworks that vary in scope, enforceability, and institutional capacity. At the global level, international organizations provide the scientific and governance foundations that shape national and regional regulatory approaches. The World Health Organization (WHO) establishes health-based standards through its drinking-water quality guidelines (GDWQ), which provide globally recognized benchmarks for chemical limits and risk-based management tools such as Water Safety Plans (Mondal et al., 2026). Complementing this, the United Nations Environment Programme (UNEP) coordinates international chemical governance through initiatives such as the Global Framework on Chemicals (GFC) and the Strategic Approach to International Chemicals Management (SAICM), promoting the safe and sustainable use of chemicals worldwide (Wong 2025). In parallel, the Food and Agriculture Organization (FAO) focuses on agricultural pollution, addressing nutrient runoff and agrochemical contamination that contribute significantly to CEC loads in water bodies (Wato 2020).

**Table 1:** Global comparison of drinking water governance for CEC using a three-criterion framework

Region/governance system	Three-criterion assessment	References
USA (Federal–state system)	*Policy & enforcement*: Federal statutes (SDWA, TSCA, FDA) exist, but regulation remains fragmented and partly reactive; private wells largely unregulated and state-level variability is high. *Technology & monitoring*: Strong research investment, UCMR monitoring, advanced treatment technologies widely adopted. *Overall capacity*: Moderate and evolving nationally, with strong performance concentrated in a few states and uneven protection across jurisdictions	USEPA (2022a, b); USEPA (2025a ); Xiao et al. (2023); Levin et al. (2024); Rosenblum et al. (2024); Smith et al. (2025); including state sources (Supplementary Table 1)
European Union (member–state system)	*Policy & enforcement*: Comprehensive and precautionary regulatory system (WFD, REACH, Directive 2020/2184) with legally binding obligations and harmonized monitoring requirements. *Technology & monitoring*: Coordinated research and prioritization via NORMAN network; chemical grouping approach under European Green Deal. *Overall capacity*: Strong, precautionary, and coordinated governance model	Puri et al. (2023); EC (2020); NORMAN network; Xabadia et al. (2021)
Australia (state/territory system)	*Policy & enforcement*: National guidelines complemented by state-level regulations; largely precautionary but often non-enforceable. *Technology & monitoring*: Strong research leadership via CRC CARE and targeted monitoring near high-risk sites. *Overall capacity*: Moderate to strong; structured and proactive but limited enforceability compared to USA and EU	NHMRC (2011); Naidu et al. (2016)
Japan (unitary centralized system)	*Policy & enforcement*: National environmental standards aligned with WHO guidelines; periodic updates and annual monitoring plans. *Technology & monitoring*: Risk-based updates and proactive monitoring supported by scientific assessments. *Overall capacity*: Strong national framework with consistent monitoring and revision mechanisms	MHLW (2008); Puri et al. (2023)
Canada (federal, provincial/territorial system)	*Policy & enforcement*: Federal drinking-water guidelines are voluntary; PFAS recently included but most CECs remain unregulated. *Technology & monitoring*: Investments in infrastructure focus mainly on microbiological contaminants. *Overall capacity*: Moderate to weak; fragmented governance and jurisdictional variability	Cook et al. (2013); Charrois (2010); GC (2022)
China (unitary centralized system)	*Policy & enforcement*: Strong regulation for traditional pollutants; emerging contaminants still in early policy development. *Technology & monitoring*: Limited monitoring infrastructure for CECs due to rapid industrialization. *Overall capacity*: Moderate but evolving; strong centralized governance with emerging policy gaps	Xu and Liu (2023); Wang et al. (2024)
Singapore (unitary city‑state system)	*Policy & enforcement*: Water security drives policy interest, but regulatory frameworks for CECs remain incomplete due to limited toxicological data. *Technology & monitoring*: High reliance on advanced treatment; monitoring constrained by data gaps. *Overall capacity*: Moderate; technologically advanced but data-limited regulatory framework	Bhagat et al. (2024); Luan (2010)
South Korea (centralized national system)	*Policy & enforcement*: Existing PFAS standards less stringent than USA; increasing calls for tighter regulations. *Technology & monitoring*: Growing research and monitoring efforts, especially related to semiconductor emissions. *Overall capacity*: Moderate; transitioning toward stronger regulation	Kim et al. (2025); National Institute of Chemical Safety, (2024)
South America (federal, decentralized and uneven systems)	*Policy & enforcement*: Limited emission standards for CECs; wastewater treatment coverage remains low. *Technology & monitoring*: Secondary treatment dominant; limited advanced treatment infrastructure. *Overall capacity*: Weak to moderate due to regulatory gaps and infrastructure limitations	Marson et al. (2022); Souza et al. (2022); Jurado-Gamez et al. (2025); Proctor et al. (2025)
African regions (varied unitary and decentralized systems)	*Policy & enforcement*: Regulations exist but enforcement is weak due to institutional and resource constraints. *Technology & monitoring*: Limited infrastructure; reliance on groundwater and private wells. *Overall capacity*: Weak; funding fragmented, and enforcement challenges are persistent	MWRWH (2015); NIS (2015); Adefolorunsho (2020); Siame et al. (2024); UNDP (2025); Matimolane (2025); WRA (2025)

Note: This table presents a comparative assessment of governance approaches for contaminants of emerging concern (CECs) across selected global jurisdictions. The three evaluation criteria: (1) policy integration, (2) regulatory instruments, and (3) implementation capacity, were applied to publicly available literature and policy documents to enable cross-jurisdictional comparison (refer to the methods section). The resulting classifications are intended to provide an indicative overview rather than an exhaustive or definitive ranking. Variations in legal frameworks, monitoring systems, data availability, and reporting practices may influence the level of detail and comparability across jurisdictions. Abbreviations: *SDWA*, Safe Drinking Water Act; *TSCA*, Toxic Substances Control Act; *FDA*, Food and Drug Administration; *UCMR*, Unregulated Contaminant Monitoring Rule; *UNDP*, United Nations Development Program; *WRA*, Water Resources Authority Kenya; *WFD*, Water Framework Directive; *REACH*, Registration, Evaluation, Authorization and Restriction of Chemicals; *EU*, European Union; *WHO*, World Health Organization; *CRC* *CARE*, Cooperative Research Centre for Contamination Assessment and Remediation of the Environment; *EC*, European Commission; *NORMAN*, Network of Reference Laboratories, Research Centres and Related Organisations for Monitoring of Emerging Environmental Substances; *NHMRC*, National Health and Medical Research Council; *NIS*, Nigerian Industrial Standard; *MHLW*, Ministry of Health, Labour and Welfare, Japan; *MWRWH*, Ministry of Water Resources, Works and Housing Government of Ghana

Among regional systems, the EU has developed one of the most comprehensive and precautionary regulatory frameworks for water quality and chemical management. The Water Framework Directive (WFD), introduced in 2000, established an integrated river basin management approach, while the Registration, Evaluation, Authorization, and Restriction of Chemicals (REACH) regulation of 2006 created a robust framework for chemical safety and risk assessment (Puri et al. 2023). The Directive 2008/105/EC established Environmental Quality Standards (EQS) for 33 Priority Substances in surface waters but excluded pharmaceutical active compounds (PhAc) (EC 2020). It also failed to incorporate EQS into river basin management plans. The framework was strengthened by Directive 2013/39/EC and updated through Directive 2020/2184, expanding the watchlist to groundwater and adding a 3-year update cycle to better address emerging contaminants. Likewise, the Groundwater Directive (GWD, Directive 2006/118/EC) established a common EQS for nitrates and agrochemicals (EC 2020; Xabadia et al. 2021).

More recently, Directive (EU) 2020/2184 on the quality of water intended for human consumption updated the EU drinking water framework in line with current scientific evidence and amended Directive 2000/60/EC. This development updated drinking-water standards to reflect new scientific evidence and identified 205 chemicals of concern, including several endocrine disruptors (EC 2020). A defining strength of the EU approach is its emphasis on coordinated research and monitoring. The NORMAN Network (https://www.norman-network.net), a European expert group dedicated to identifying and prioritizing emerging contaminants, has played a critical role in developing monitoring tools and supporting evidence-based policymaking.

France has already incorporated NORMAN tools into national river basin management planning, demonstrating how coordinated scientific infrastructure can inform policy and improve surveillance of emerging substances (Dulio et al. 2018). Meanwhile, in the Netherlands and Belgium, the Dutch Ministry of Infrastructure and Water Management and the Public Waste Agency of Flanders (OVAM) collect and analyze data on CECs such as PFOS and PFOA to regulate and monitor environmental impacts and uphold environmental policies (Puri et al. 2023). Recently, the EU launched the European Green Deal, which aims to achieve carbon neutrality by 2050 (EC 2020). It represents a further evolution of EU chemical policy by shifting regulatory focus from individual substances to broader chemical groupings. This transition is particularly important for large chemical classes such as PFAS, pharmaceuticals, and agrochemicals, where traditional risk assessment approaches struggle to address mixture effects and the sheer scale of chemical inventories. The shift reflects the precautionary principle, which advocates protective action even when scientific evidence is incomplete, particularly when potential impacts may be irreversible (EC 2020; Xabadia et al. 2021). The EU has positioned itself as a global leader in precautionary chemical governance by emphasizing prevention, group-based regulation, and long-term sustainability.

In contrast, the US has developed a highly research-intensive but more decentralized regulatory system. The USEPA regulates drinking-water contaminants under the SDWA, setting quality standards for more than 98 pollutants and gathering input from experts and the public to identify emerging hazards (USEPA 2022b). Additional oversight is provided by the Toxic Substances Control Act (TSCA) and the Food and Drug Administration (FDA), which evaluate environmental risks associated with new pharmaceuticals and chemicals to ensure that their concentrations in surface waters do not exceed 1 ppb upon introduction (USEPA 2025a). The USA has also invested heavily in research and data infrastructure. Federal agencies such as the National Institutes of Health (NIH), Department of Energy (DOE), and Department of Agriculture (USDA) support environmental research and sustainability assessments that inform policy development (Sarker 2025). The USEPA’s Enforcement and Compliance History Online (ECHO) platform exemplifies the use of management information systems to enhance transparency, pollution tracking, and regulatory compliance (Sarker 2025). Unlike the centralized European model, however, US research and monitoring efforts are distributed across multiple agencies, reflecting a decentralized governance structure (Refer to Table 1).

Equally important, state-level initiatives also influence the US regulatory landscape (refer to Fig. 3 and Supplementary Table 1). For instance, California’s State Water Resources Control Board oversees monitoring programs for CEC risks associated with water recycling (Anderson et al. 2010). This federal-state arrangement offers flexibility and encourages innovation but can lead to regulatory variation. Overall, the US system blends legally binding federal standards with state-level efforts to enforce stricter rules, resulting in a dynamic yet occasionally fragmented policy environment.

Several other developed economies have established structured regulatory frameworks that share similarities with either the EU or the US models. In Japan (refer to Table 1), national environmental policy sets quality standards for air, soil, and water contamination, covering 26 priority substances (MHLW 2008). In 2002, drinking water quality standards were revised to align with WHO guidelines, establishing standards for 51 health-related chemicals and introducing annual monitoring plans and periodic updates based on new scientific evidence (MHLW 2008; Puri et al. 2023). This proactive monitoring and revision process reflects a strong commitment to evidence-based policy.

Australia’s regulatory framework closely parallels the US federal–state model. The Australian Drinking Water Guidelines (ADWG) establish national health-based standards, while states implement complementary regulations such as Queensland’s Environmental Protection (Water) Policy and Victoria’s Pollution of Waters by Oil and Noxious Substances Act (Naidu et al. 2016; NHMRC 2011). The Cooperative Research Centre for Contamination Assessment and Remediation of the Environment (CRC CARE) serves as a national research hub, supporting guideline development and remediation efforts for priority contaminants such as PFAS. Differences in enforceability are particularly evident in PFAS regulation. Australia has historically recommended guideline values around 70 ng/L for PFOS and PFOA (NHMRC 2011), while the USA has issued extremely low health advisory levels of 0.004 ng/L for PFOA and 0.020 ng/L for PFOS, though enforceable federal standards remain under development (Cotruvo et al. 2023). State-level standards in the USA range widely from 13 to 1000 ng/L, illustrating the decentralized nature of risk management (USEPA 2025a). Monitoring strategies also differ, as Australia relies on targeted monitoring near high-risk sites, whereas the US UCMR provides nationwide occurrence data (USEPA 2021a). However, both countries employ advanced treatment technologies such as granular activated carbon, reverse osmosis, and advanced oxidation processes.

Canada, by contrast, faces significant regulatory gaps. Drinking water quality falls under provincial or territorial jurisdiction, but like in the USA, these regulations generally apply only to public systems (Cook et al. 2013). Approximately four million Canadians who rely on private wells are responsible for their own testing and monitoring (Charrois 2010). While government agencies provide advice and guidelines, there is typically no legal requirement for homeowners to comply with testing recommendations for existing wells. Some provinces have stricter rules for newly constructed wells (GC 2022). In New Brunswick, the Potable Water Regulation requires that all newly constructed wells be tested for bacteria and inorganic contaminants such as arsenic before use (Cook et al. 2013; Dunn et al. 2014). Although PFAS were included in drinking water guidelines in 2022, many other CECs, particularly pharmaceuticals and personal care products, remain unregulated (GC 2022). While investments in water infrastructure have reduced long-term drinking-water advisories in indigenous communities, these initiatives primarily target microbiological contaminants rather than CECs (GC 2021).

On the other hand, emerging-economy water policy reflects the tension between rapid industrial growth and still-developing regulation of CECs. In China, regulations are well established for traditional pollutants such as heavy metals and industrial effluents, but governance of pharmaceuticals, personal care products, microplastics, and some PFAS-related compounds remains at an earlier stage (Wang et al. 2024). This gap is linked to rapid industrial expansion, uneven monitoring capacity, and limited standardized methods for long-term CEC surveillance, leaving many emerging substances insufficiently categorized and regulated at the national level (Wang et al. 2024; Xu & Liu 2023). Similarly, Singapore faces unique pressures due to its small, densely populated urban environment. As a city-state that depends heavily on imported water and advanced treatment systems like NEWater and desalination, even minimal releases of pharmaceuticals, personal care products, and microplastics are seen as a concern for water security (Bhagat et al. 2024; Luan 2010). The limited space and urban land-use pressures increase the risk of contaminant recycling. However, regulations for many emerging compounds still lag behind scientific findings, which hampers the implementation of stricter standards (Bhagat et al. 2024).

In comparison, South Korea increasingly demonstrates how industrial concentration can drive both risk and regulatory change. Emissions from the semiconductor and advanced manufacturing sectors have accelerated policy action on PFAS and other emerging contaminants. Drinking-water guidance now includes provisional PFAS thresholds of 70 ng/L for PFOA and PFOS (individually or combined) and 480 ng/L for PFHxS, less stringent than US federal limits but a clear step toward tighter oversight (Kim et al. 2025; NICS 2024). PFAS have also been designated as priority-controlled substances under the Persistent Organic Pollutants Control Act, reflecting growing concern about persistence, bioaccumulation, and health risks, with expanded monitoring led by the National Institute of Chemical Safety (NICS 2024).

In South America, governance of CECs remains fragmented and limited. Dedicated emission standards and drinking‑water limits for pharmaceuticals, agrochemicals, and PFAS are still scarce, and monitoring programs are only beginning to emerge (Souza et al. 2022). Existing regulations primarily address conventional pollutants. For example, Brazil’s national environment council, or Conselho Nacional do Meio Ambiente (CONAMA), Resolution 357 covers selected agrochemicals but not the broader range of CECs now detected in water resources (Marson et al. 2022; Souza et al. 2022). In Colombia, 117 emerging pharmaceutical contaminants have been reported in major watersheds, yet regulatory frameworks and monitoring are still insufficient to manage these substances systematically (Jurado-Gamez et al. 2025; María Eugenia Buitrago-González et al., 2024). In Argentina, a recent survey of wastewater treatment plants found 74 CECs in liquid effluents, highlighting the widespread contamination and the lack of specific drinking water or emission standards for these substances (Proctor et al. 2025). Nonetheless, the country’s regulatory framework primarily depends on traditional water-quality parameters and does not currently include substance-specific standards for these CECs. Therefore, limited standards, weak enforcement, and underdeveloped treatment capacity together increase vulnerability to unmonitored exposure.

In the same vein, in many African countries, regulatory challenges are closely tied to infrastructure limitations, rapid urbanization, and institutional capacity constraints (refer to Table 1). Many communities rely on groundwater and private wells due to limited public water supply systems (Adefolorunsho 2020; Siame et al. 2024). In contrast, in the USA, private wells serving fewer than 25 people are not regulated under the SDWA, leaving millions responsible for their own water testing (Xabadia et al. 2021). Similar gaps exist in Canada for private water supplies (Charrois 2010). Moving forward, although legislation exists in countries such as South Africa, Ghana, Kenya, and Nigeria, enforcement remains inconsistent (Matimolane 2025; MWRWH 2015; NIS 2015; WRA, 2025). Agricultural runoff, industrial waste, and e-waste disposal further exacerbate contamination risks (Nwokediegwu et al. 2024). Moreover, funding for water and contamination management is often embedded within broader development programs supported by the World Bank, Green Climate Fund, and United Nations Development Program (Lozano-Gracia & Soppelsa 2019; UNDP 2025). Collectively, these cross-cutting challenges persist globally. Communication barriers continue to hinder policy development, as technical scientific language often limits engagement by policymakers and communities (Gani et al. 2021), and climate change further complicates water quality management by increasing pollutant transport and harmful algal blooms (HABs) driven by extreme weather and agricultural runoff (Ahmed et al. 2016; Brooks et al. 2016).

All in all, global policy approaches to CEC management reflect a spectrum of regulatory maturity. The EU demonstrates coordinated and precautionary governance. The USA blends robust federal standards with decentralized implementation. However, despite this progress and variety of approaches, current policies remain reactive and insufficient to effectively tackle emerging water contamination challenges. Australia and Japan maintain structured but less enforceable systems, and Canada, emerging economies, and developing regions face institutional, regulatory, and resource constraints. Overall, while progress is evident, the global policy landscape remains fragmented. Therefore, strengthening coordination, improving monitoring, and enhancing communication between science and policy will be essential to effectively address the growing challenges posed by CECs.

## Recommendations

According to Dulio et al. (2018), Xabadia et al. (2021), and Dettori et al. (2022), safeguarding the long-term safety of drinking water from CECs requires a coordinated, anticipatory, equitable, and evidence-based policy response. The first step is to develop a phased national roadmap for CEC management under the SDWA. Our proposed roadmap builds on USEPA’s PFAS Strategic Roadmap by establishing multi-CEC class-based governance and precautionary benchmarks for unregulated contaminants (USEPA 2021c). In the first term (1–3 years), this roadmap should mandate baseline monitoring of priority CEC classes, including PFAS, pharmaceuticals, and agrochemicals, across all water systems, including small, rural, and tribal utilities that are frequently excluded from comprehensive surveillance; for PFAS specifically, the rationale is not that no regulation exists, but that current federal standards address only selected compounds within a much larger chemical class, making broader class-based monitoring and governance necessary (Dulio et al. 2018; USEPA 2021c, 2025f). Over 3–7 years, precautionary health-based benchmarks should be introduced to allow standards to adapt with advances in toxicology and exposure science. In the longer term (7–15 years), policies should shift from regulating individual chemicals toward class-based governance and prioritized cumulative-risk approaches that better reflect real-world co-exposures. This does not imply that regulators can test every possible chemical mixture or concentration scenario; rather, current science supports grouping and prioritizing chemicals with shared structures, mechanisms, or exposure pathways because the number of possible combinations is effectively too large for exhaustive testing, and whole-mixture data remain limited (Braeuning et al. 2022; Rider 2022).

A second recommendation is to shift the financial responsibility for CEC management from water consumers to chemical producers. Currently, communities bear a disproportionate burden because treatment costs are funded by ratepayers, and environmental costs are not fully accounted for. To avoid reproducing CERCLA’s historical weaknesses, such a system should include a protected industry-funded revenue stream and a stronger administrative cost-recovery process, so agencies are not forced to rely primarily on lengthy litigation to secure cleanup funds (GAO 2009, 2025; USDJ 2024). Therefore, expanding federal cost-recovery methods, based on CERCLA principles, to cover widespread CEC contamination could help address this imbalance (USEPA 2025a). In addition, implementing an EPR system for high-risk chemical groups could provide ongoing funding for monitoring, treatment upgrades, and cleanup efforts, while also encouraging the development of safer chemicals upstream (OECD 2016).

The final recommendation is to invest in advanced treatment technologies while ensuring clear commitments to equity and transparency (Anderson et al. 2010; Siame et al. 2024). Although methods such as ion exchange, nanofiltration, and hybrid adsorption are highly effective at removing contaminants, their adoption varies across utilities. In this context, ongoing compliance monitoring tools such as the USEPA’s Environmental Compliance History Online (ECHO) PFAS Analytics can complement policy evaluations by improving visibility into implementation performance and system-level effectiveness (USEPA 2021c). However, support through federally funded demonstration projects and ongoing technical assistance for under-resourced systems can help reduce disparities. Furthermore, establishing a national, publicly accessible CEC disclosure platform, such as the USEPA Toxics Release Inventory, would enhance accountability, facilitate community engagement, and highlight populations currently overlooked in water governance.

## Conclusion

All in all, our study provides a policy-focused assessment of how US public water systems handle emerging contaminants, revealing notable differences in regulatory capacity among states. Although the analysis relies on publicly available policy documents and program data, which may underrepresent informal efforts, recent regulatory changes, or actual implementation success, it provides a transparent, reproducible framework for evaluating governance readiness at scale. Recent federal regulatory developments, including proposed PFAS drinking water standards and other actions on CECs, further underscore the timeliness of the state-level gaps and policy considerations identified in this study. Importantly, the findings show that having policies alone does not ensure comprehensive protection, particularly when actions target only certain contaminant categories. Future research should include long-term policy monitoring, compliance data, and health or exposure information to better evaluate regulatory effectiveness over time. Additionally, adding data on funding, treatment performance, and community impacts would further support evidence-based decisions. Despite these limitations, our study establishes a crucial policy foundation for identifying gaps, informing targeted strategies, and promoting more equitable and resilient water governance across the USA.

## Data Availability

All essential data supporting this study are in the manuscript or supplementary materials.
